# Supplementary material for: Decent Work, ILO’s Response to the Globalization of Working Life: Basic Concepts and Global Implementation with Special Reference to Occupational Health
Source: Int J Environ Res Public Health. 2020 May 12;17(10):3351. doi: 10.3390/ijerph17103351 (PMC7277660; doi:10.3390/ijerph17103351)
Supplement: Supplementary file 1 [file ijerph-17-03351-s001.pdf]

Supplementary 1. ILO and UN Documents analysed for this article.

International Labour Organization. ILO Declaration on Fundamental Principles and Rights at Work. International Labour Office, Geneva 1998. Available online: https://www.ilo.org/declaration/lang--en/index.htm (accessed on 30 July 2019). (2).

United Nations. Secretary-General proposes Global Compact on Human Rights, Labour, Environment. The address of Secretary-General Kofi Annan to the World Economic Forum in Davos, Switzerland, on 31 January 1999. United Nations 1999. Available online: https://www.un.org/press/en/1999/19990201.sgsm6881.html (accessed on 30 July 2019).(3).

International Labour Organization. Decent Work, Report of the Director-General, International Labour Conference, 87th Session, International Labour Office, Geneva 1999. Available ohttps://www.ilo.org/public/libdoc/ilo/P/09605/09605(1999-87).pdf (accessed on 30 July 2019). (4).

nternational Labour Organization. Address by Mr. Juan Somavia, Director General of the International Labour Organization, 1 June 1999, International Labour Conference, 87th Session, Geneva 1999. Available online: https://www.ilo.org/public/english/standards/relm/ilc/ilc87/rep-i.htm (accessed on 30 July 2019). (5).

International Labour Organization. ILO Declaration on Social Justice for a Fair Globalization. International Labour Office, Geneva 2008. Available online:http://www.ilo.org/public/libdoc/ilo/2008/108B09_147_engl.pdf (accessed on 30 July 2019).(6).

United Nations. The Millennium Development Goals Report 2015. United Nations New York 2015. Available online: https://www.un.org/millenniumgoals/2015_MDG_Report/pdf/MDG%202015%20rev%20(July%201.pdf (accessed on 30 July 2019).(7).

United Nations. Transforming our world: the 2030 Agenda for Sustainable Development. United Nations 2015. Available online: https://sustainabledevelopment.un.org/post2015/transformingourworld (accessed on 30 July 2019).(8).

International Labour Organization. Decent Work Indicators in the SDGs. Global Indicator Framework. ILO Department of Statistics and the ILO Regional Office for Asia and the Pacific 2016. Available online: https://www.ilo.org/wcmsp5/groups/public/---asia/---ro-bangkok/documents/presentation/wcms_493329.pdf (accessed on 30 July 2019).(9).

International Labour Organization. Decent Work Country Programmes. International Labour Office 1999. Available online: https://www.ilo.org/global/about-the-ilo/how-the-ilo-works/departments-and-offices/ program/dwcp/lang--en/index.htm (accessed on 30 July 2019).(10).

International Labour Organization. ILO Implementation Plan 2030 Agenda for Sustainable Development 2016. CABIN-160421-2-EN.docx. Available online: https://www.ilo.org/wcmsp5/groups/public/---dgreports/---dcomm/---webdev/documents/publication/wcms_510122.pdf (accessed on 30 July 2019).(11).

International Labour Organization. What is Decent Work? 2016. Available online: https://www.youtube. com/watch?time_continue=110&v=mZpyJwevPqc (accessed on 30 July 2019).(12).

International Labour Organization. Decent Work Country Diagnostics. Technical guidelines to drafting diagnostic report. International Labour Office, Geneva 2015. Available online: https://www.ilo.org /wcmsp5/groups/public/---ed_mas/---program/documents/genericdocument/wcms_561044.pdf (accessed on 31 July 2019). (13).

International Labour Organization. ILO Decent Work Country Programme. Practical guidebook, Version 4. First published, 2005. International Labour Office, Geneva, 2016. Available online: https://www.ilo.org /wcmsp5/groups/public/---ed_mas/---program/documents/genericdocument/wcms_561025.pdf (accessed on 28 August 2019).(14).

International Labour Organization. Improving safety and health at work through a Decent Work Agenda. International Labour Office 2010. Available online: https://www.ilo.org/global/topics/safety-and-health-at-work/programmes-projects/WCMS_149466/lang--en/index.htm (accessed on 30 July 2019).(15)

International Labour Organization Decent Work website. Discussions of high-level evaluations: Strategies and Decent Work Country Programmes. Available online: https://www.ilo.org/global/topics/decent-work/lang--en/index.htm (accessed on 28 August 2019).(16)

International Labour Organization. Status of Decent Work Country Programme Development by Region. International Labour Office 2019. Available online: https://www.ilo.org/wcmsp5/groups/public /@ed_mas/@program/documents/genericdocument/wcms_630738.pdf (accessed on 28 August 2019).(17).

International Labour Organization. ILO Decent Work Country Programmes. International Labour Office 2019. Available online: https://www.ilo.org/global/about-the-ilo/how-the-ilo-works/departments-and-offices/program/dwcp/lang--en/index.htm (accessed on 28 August 2019).(18).

International Labour Organization. ILO Constitution. International Labour Organization 1919. Available online: https://www.ilo.org/dyn/normlex/en/f?p=1000:62:0::NO:62:P62_LIST_ENTRIE_ID:2453907:NO#A1 (accessed on 30 July 2019).(19).

International Labour Organization. ILO Centenary Declaration for the Future of Work. 2019. Available online: https://www.ilo.org/tokyo/WCMS_711674/lang--en/index.htm (accessed on 30 July 2019).(20).

International Labour Organization. Global Strategy on Occupational Safety and Health. Conclusions adopted by the International Labour Conference at its 91st Session, 2003. International Labour Office 2004. Available online: https://www.ilo.org/wcmsp5/groups/public/---ed_protect/---protrav/---safework/ documents/meetingdocument/wcms_111293.pdf (accessed on 30 July 2019).(21).

International Labour Organization. Convention No.161 on Occupational Health Services. International Labour Office, Geneva 1985. Available online: https://www.ilo.org/dyn/normlex/en/f?p=NORM LEXPUB:12100:0::NO::P12100_ILO_CODE:C161 (accessed on 28 August 2019).(22).

International Labour Organization. Recommendation No. 171 on Occupational Health Services. International Labour Office, Geneva 1985. Available online: https://www.ilo.org/dyn/normlex/en/f?p= NORMLEXPUB:12100:0::NO::P12100_ILO_CODE:R171 (accessed on 28 August 2019).(23).

International Labour Organization. Convention No. 187 on Promotional Framework for Occupational Safety and Health. International Labour Office, Geneva 2006. Available online: https://www.ilo.org/dyn/normlex/ en/f?p=NORMLEXPUB:12100:0::NO::P12100_ILO_CODE:C187 (accessed on 30 July 2019).(26).

International Labour Organization. Women and men in the informal economy: a statistical picture. Third edition. International Labour Office, Geneva 2018. Available online: https://www.ilo.org/wcmsp5/groups/ public/---dgreports/---dcomm/documents/publication/wcms_626831.pdf (accessed on 28 August 2019).(29).

United Nations. Global health and foreign policy. Sixty-seventh session Agenda item 123. Resolution adopted by the General Assembly on 12 December 2012 [without reference to a Main Committee (A/67/L.36 and Add.1)] 67/81. Available online: https://www.un.org/en/ga/search/view_doc.asp?symbol=A/RES/ 67/81 (accessed on 15 August 2019). (34)

United Nations. UN High-Level Meeting on universal health coverage. 2019. Available online: https://www.un.org/pga/73/wp-content/uploads/sites/53/2019/07/FINAL-draft-UHC-Political-Declaration.pdf (accessed on 4 October 2019).(35).

World Health Organization. WHA72.4. Preparation for the high-level meeting of the United Nations General Assembly on universal health coverage. World Health Organization, Geneva 2019. Available online: https://apps.who.int/gb/ebwha/pdf_files/WHA72/A72_R4-en.pdf (accessed on 15 August 2019).(38).

International Labour Organization. Work for a brighter future: global commission on the future of work. International Labour Office, Geneva 2019. Available online: https://www.ilo.org/wcmsp5/groups/public/---dgreports/---cabinet/documents/publication/wcms_662410.pdf (accessed 13 July 2019). (46)

International Labour Organization. Women and men in the informal economy: a statistical picture. Third edition. International Labour Office, Geneva 2018. Available online: https://www.ilo.org/wcmsp5/groups/ public/---dgreports/---dcomm/documents/publication/wcms_626831.pdf (accessed 13 July 2019). (54).

International Labour Organization. Independent Evaluation of the ILO's Decent Work Country Programmes, strategies and actions in the Caribbean (2010–2015). International Labour Office, Geneva 2015. Available online: https://www.ilo.org/wcmsp5/groups/public/---ed_mas/---eval/documents/publication/wcms_419 437.pdf (accessed on 22 January 2020). (62)

International Labour Organization. Independent evaluation of the ILO's Decent Work Country Programme Strategies and Actions in the Western Balkans 2012–2015. International Labour Office, Geneva 2016. Available online: https://www.ilo.org/wcmsp5/groups/public/---ed_mas/---eval/documents/publication /wcms_532848.pdf (accessed on 22 January 2020).(63).

International Labour Organization. Independent evaluation of the ILO's Decent Work Country Programme strategies and actions in the Mekong subregion 2012–2017. International Labour Office, Geneva 2017. Available online: https://www.ilo.org/wcmsp5/groups/public/---ed_mas/---eval/documents/publication /wcms_583706.pdf (accessed on 22 January 2020).(64).

International Labour Organization. Independent evaluation. ILO's Programme of Work in Four Selected Member Countries of the Southern African Development Community (SADC) (Lesotho, Madagascar, South Africa and the United Republic of Tanzania), 2014–2018. International Labour

Office, Geneva 2019. Available online: https://www.ilo.org/wcmsp5/groups/public/---ed_mas/---eval/documents/publication/wcms_722 182.pdf (accessed on 22 January 2020). (65).

International Labour Organization. Time to Act for SDG 8 Integrating Decent Work, Sustained Growth and Environmental Integrity. International Labour Office, Geneva 2019. Available online: https://www.ilo.org/wcmsp5/groups/public/---dgreports/---inst/documents/publication/wcms_712685. pdf (accessed on 22 March 2020).(66)

International Labour Organization. COVID-19 and the world of work. Available online: https://www.ilo.org/global/topics/coronavirus/lang--en/index.htm (accessed on 22 March 2020).(67)

International Labour Organization. COVID-19 pandemic. Almost 25 million jobs could be lost worldwide as a result of COVID-19. Available online: https://www.ilo.org/global/about-the-ilo/newsroom/news/ WCMS_738742/lang--en/index.htm (accessed on 22 March 2020). (68).

Supplementary 2. ILO DWCP independent evaluations in 2006–2018.

Country or sub-region	Report	Available Online
The Philippines 2006	Independent Evaluation of the ILO's Country Programme to the Philippines: 2000–2005	https://www.ilo.org/wcmsp5/groups/public/---ed_mas/---eval/documents/publication/wcms_083426.pdf
Argentina 2007	Independent Evaluation of the ILO's Country Programme for Argentina: 2001–2006	https://www.ilo.org/wcmsp5/groups/public/---ed_mas/---eval/documents/publication/wcms_087338.pdf
Ukraine 2007	Independent Evaluation of the ILO's Country Programme to Ukraine: 2000–2006	https://www.ilo.org/wcmsp5/groups/public/---ed_mas/---eval/documents/publication/wcms_087341.pdf
Zambia 2008	Independent Evaluation of the ILO's Country Programme for Zambia: 2001–2007	https://www.ilo.org/wcmsp5/groups/public/---ed_mas/---eval/documents/publication/wcms_099531.pdf
Jordan 2008	Independent Evaluation of the ILO's Country Programme for the Hashemite Kingdom of Jordan: 2002–2007	https://www.ilo.org/wcmsp5/groups/public/---ed_mas/---eval/documents/publication/wcms_099572.pdf
Indonesia 2009	Independent Evaluation of the ILO's Decent Work Country Programme for Indonesia: 2006–2009	https://www.ilo.org/wcmsp5/groups/public/---ed_mas/---eval/documents/publication/wcms_116334.pdf
Kyrgyzstan 2010	Independent Evaluation of the ILO's Decent Work Country Programme for Kyrgyzstan: 2006–2009	https://www.ilo.org/wcmsp5/groups/public/---ed_mas/---eval/documents/publication/wcms_146035.pdf
Tanzania 2010	Independent Evaluation of the ILO's Country Programme for the United Republic of Tanzania 2004–2010	https://www.ilo.org/wcmsp5/groups/public/---ed_mas/---eval/documents/publication/wcms_146037.pdf
Bahia, Brazil 2011	Independent evaluation of the ILO's support to the Bahia Decent Work Agenda: 2008–2010 (Brazil)	https://www.ilo.org/wcmsp5/groups/public/---ed_mas/---eval/documents/publication/wcms_165832.pdf
India 2012	Independent Evaluation of the ILO's Decent Work Country Programme for India: 2007–2012	https://www.ilo.org/wcmsp5/groups/public/---ed_mas/---eval/documents/publication/wcms_191678.pdf
Arab Region 2013	Independent evaluation of the ILO's strategy to promote decent work in the Arab region: A cluster evaluation of	https://www.ilo.org/wcmsp5/groups/public/---ed_mas/---eval/documents/publication/wcms_226356.pdf

	Jordan, Lebanon and the Occupied Palestinian Territory, 2008–2012	
North Africa 2014	Independent evaluation of the ILO's Decent Work Country Programme Strategies and Activities in North Africa 2010–2013	https://www.ilo.org/wcmsp5/groups/public/---ed_mas/---eval/documents/publication/wcms_314439.pdf<br><br>https://www.ilo.org/wcmsp5/groups/public/---ed_mas/---eval/documents/publication/wcms_316825.pdf
The Caribbean 2015	Independent evaluation of the ILO's Decent Work Country Programmes, strategies and actions in the Caribbean 2010–2015	https://www.ilo.org/wcmsp5/groups/public/---ed_mas/---eval/documents/publication/wcms_419437.pdf
Western Balkans 2016	Independent evaluation of the ILO's Decent Work Country Programmes, strategies and actions in the Western Balkans (2012–2015)	https://www.ilo.org/wcmsp5/groups/public/---ed_mas/---eval/documents/publication/wcms_532848.pdf
Mekong sub-region 2017	Independent evaluation of the ILO's Decent Work Country Programme strategies and actions in the Mekong subregion, 2012–2017	https://www.ilo.org/wcmsp5/groups/public/---ed_mas/---eval/documents/publication/wcms_583706.pdf
Lebanon and Jordan 2018	Independent evaluation of ILO's programme of work in Lebanon and Jordan in Terms of Decent Work and the Response to the Syrian Refugee Crisis, 2014–2018	https://www.ilo.org/wcmsp5/groups/public/---ed_mas/---eval/documents/publication/wcms_646718.pdf
